# Impact of the 2012 extreme drought conditions on private well owners in the United States, a qualitative analysis

**DOI:** 10.1186/s12889-016-3039-4

**Published:** 2016-05-24

**Authors:** Michelle Murti, Ellen Yard, Rachel Kramer, Dirk Haselow, Mike Mettler, Rocky McElvany, Colleen Martin

**Affiliations:** Epidemic Intelligence Service, Centers for Disease Control and Prevention, Atlanta, GA USA; Centers for Disease Control and Prevention, Health Studies Branch, Atlanta, GA USA; SciMetrika LLC, Durham, NC USA; Arkansas Department of Health, Little Rock, AR USA; Indiana State Department of Health, Indianapolis, IN USA; Oklahoma State Department of Health, Oklahoma City, OK USA

**Keywords:** Climate, Droughts, Qualitative research, Water quality, Water wells

## Abstract

**Background:**

Extreme hot and dry weather during summer 2012 resulted in some of the most devastating drought conditions in the last half-century in the United States (U.S.). While public drinking water systems have contingency plans and access to alternative resources to maintain supply for their customers during drought, little is known about the impacts of drought on private well owners, who are responsible for maintaining their own water supply. The purpose of this investigation was to explore the public health impacts of the 2012 drought on private well owners’ water quality and quantity, identify their needs for planning and preparing for drought, and to explore their knowledge, attitudes, and well maintenance behaviors during drought.

**Methods:**

In the spring of 2013, we conducted six focus group discussions with private well owners in Arkansas, Indiana, and Oklahoma.

**Results:**

There were a total of 41 participants, two-thirds of whom were men aged 55 years or older. While participants agreed that 2012 was the worst drought in memory, few experienced direct impacts on their water quantity or quality. However, all groups had heard of areas or individuals whose wells had run dry. Participants conserved water by reducing their indoor and outdoor consumption, but they had few suggestions on additional ways to conserve, and they raised concerns about limiting water use too much. Participants wanted information on how to test their well and any water quality issues in their area.

**Conclusions:**

This investigation identified information needs regarding drought preparedness and well management for well owners.

## Background

During the summer of 2012, hot and dry conditions caused some of the worst drought conditions in the last half-century for many parts of the United States (U.S.) [1]. The U.S. Drought Monitor reported ‘Extreme’ and ‘Exceptional’ drought conditions across several states in the West, Midwest, and Great Plains regions [2]. The National Oceanic and Atmospheric Administration (NOAA) reported 123 deaths directly related to the extreme heat associated with the summer of 2012 [3]. The extreme heat and dry conditions also had significant impacts on the agricultural sector. Half of the counties in the U.S. had drought-related disaster declarations [4]. News reports on private well owners and drilling companies identified significant increases and concerns associated with wells running dry across the Midwest [5].

Drought has been defined as “a temporary lack of water, which is, at least partly, caused by abnormal climate conditions and is damaging to an activity, group, or the environment” [6]. Many measures have been used to describe drought; however, the U.S. Drought Monitor provides the most comprehensive summary of drought in the U.S., producing weekly reports and maps indicating drought intensity according to five levels: Abnormally Dry, Moderate, Severe, Extreme, and Exceptional [7].

Unlike other natural disasters, drought has a slower onset and longer timespan—often occurring over months to years—which creates challenges for measuring its impact [6, 8]. The majority of drought research from developed countries has focused on the coping and mental health consequences from loss of agriculture and its associated socioeconomic decline [8–11]. The personal, community, and public health impacts of drought are not as well described. In 2010, the U.S. Centers for Disease Control and Prevention (CDC), in partnership with the U.S. Environmental Protection Agency (EPA), NOAA, and the American Water Works Association (AWWA), released its review of the public health impacts of drought in the report, *When every drop counts: protecting public health during drought conditions* [12]. The review postulated that drought impacts domestic potable water supplies by impairing both the quantity and quality of surface and groundwater resources. Stagnant, shallow surface waters have increased concentrations of pollutants and higher sediment loads, and shallow groundwater systems are susceptible to intrusion from surface pollutants.

Drought-prone regions often have planning, preparedness, and response options for mitigating and adapting to drought. Several local and state agencies have developed long-range plans for coping with drought [13]. These strategies attempt to ensure a safe and adequate water supply for consumers during drought. However, local complexities in water rights, public versus private systems, and water conservation policies can create challenges when implementing these plans [14–16]. Most of these plans do not specifically include drought planning for private well owners. Individual households that rely on private well water are responsible for managing their own water supply; thus, they could be more adversely affected during drought. Furthermore, research on the effectiveness of water conservation strategies has primarily focused on customers of community water systems, to the exclusion of private well owners [17–20].

Approximately 11 % of the U.S. population rely on private wells for drinking water [21]. In a 2009 report, the U.S. Geological Survey (USGS) identified areas of concern for private well owners from groundwater contamination, and recommended the need for private well owner education on water testing, treatment, and other issues [22]. Studies of private well owners have also identified gaps and barriers in homeowners’ routine testing of wells, and associated adverse water quality [23–27].

Given known concerns about well stewardship and the lack of their inclusion in water conservation or drought response, we hypothesized that private well owners may be adversely and differentially impacted by decreased water quality and quantity during a drought. News reports during the summer of 2012 suggested there were concerns for public health impacts for private well owners whose wells had gone dry [28, 29]. The gaps in our understanding of the behaviors and practices of well owners, both generally and during a drought, prompted the Health Studies Branch of the National Center for Environmental Health at CDC to partner with Arkansas (AR), Indiana (IN) and Oklahoma (OK) to investigate the public health impacts of drought on domestic use private well owners in terms of water quality and quantity; explore their knowledge, attitudes and behaviors regarding well maintenance during drought; and identify their needs for planning and preparing for drought.

## Methods

**Fig. 1:**
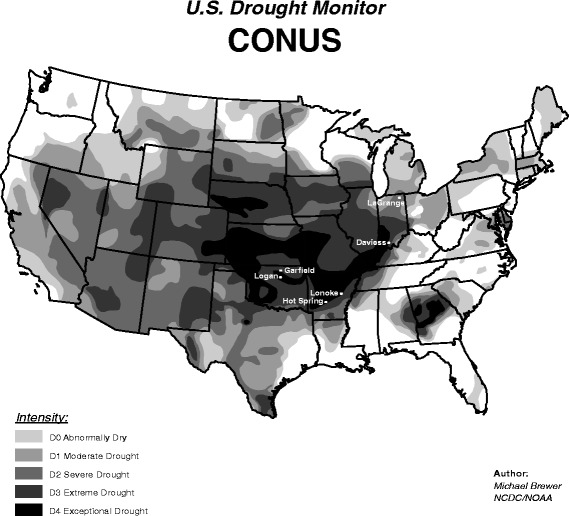
Locations of focus groups in relationship to areas of drought in the Contiguous U.S. (CONUS) as of August 7 2012. * * Reprinted with permission of the U.S. Drought Monitor and its partners: the National Drought Mitigation Center, the United States Department of Agriculture, and the National Oceanic and Atmospheric Administration

In spring 2013, we conducted six focus groups with a research team, study design and analysis methodology guided by the consolidated criteria for reporting qualitative research (COREQ) [30]. Participants were English-speaking adults (≥18 years of age) who were currently using a private well for their domestic (household) water and had used a private well for the past 12 months. Focus groups were conducted in the counties of Hot Spring and Lonoke, AR; LaGrange and Daviess, IN; and Logan and Garfield, OK. These states were selected due to their drought conditions, geographic diversity, and range in their density of private well use. Figure 1 shows the location of the focus groups (AR1, AR2, IN1, IN2, OK1 and OK2) in relation to drought levels as of August 7, 2012; during the summer of 2012, AR, IN and OK had most of their states in ‘extreme’ or ‘exceptional’ levels of drought [2]. According to the 1990 U.S. Census, private well ownership ranged from approximately 15 % in OK, to 20 % in AR and 27 % in IN [31]. We selected two counties in each state for the locations of the focus groups based on well owner density, areas of drought, and ideally representing two different aquifer systems and different socio-demographic characteristics in the counties.

This study was deemed to be exempt from human subjects research. We developed a pre-focus group questionnaire to gather information on household demographics, water use, and well testing experience of the participants, and a moderator guide for the sessions that consisted of structured questions, probes, and follow-up questions.

We also developed promotional material (newspaper notices and local flyers) for distribution through county level public health representatives. A market research and consulting firm recruited participants by random dialing from county telephone lists and through referrals from neighbors or family members who had been contacted or recruited. A total of 11 to 14 eligible participants were recruited per focus group session with the intention of final focus group sizes of 8 to 10 individuals. Participants were mailed the pre-focus group questionnaire in advance and received a reminder call the day before the session. Participants provided informed consent at the start of their session and were provided state-specific information packages with local, state and national resources for well owners at the end of their session.

The pre-focus group questionnaire and moderator guide were nearly identical across focus groups, with one exception. In Oklahoma, the state’s water agency (the Oklahoma Water Resources Board, OWRB), had recently completed their Comprehensive Water Plan and had conducted several community engagement sessions to inform participants of the Comprehensive Water Plan [32]. Thus, OK1 and OK2 participants were asked in their pre-focus group questionnaire whether they had heard of the state Water Plan or had participated in a state water planning activity, such as a town hall meeting.

A trained facilitator led the focus group discussions using the moderator guide. Questions explored participants’ experiences with the drought, changes to their water quality and quantity during the drought, their responses to the drought, and adherence to private well water testing guidelines. Participants were also asked to describe their private well system (e.g., depth, age of well, number of wells) to assess their level of knowledge about their system, and identify differences amongst participants’ well systems that may impact their water quality and quantity. Systematic procedures were used to enhance data capture and improve the reliability and validity of the data collected including: i) audio-taping and professional transcription of sessions; ii) inclusion of notes taken by three silent observers from the investigation team; iii) immediate debriefing after sessions with the moderator and observers; and iv) reviewing high level themes with participants at the end of each focus group. The purpose of considering the perspectives of the research team and the participants in the interpretation of data was to limit bias and reach consensual validation of substantive significance of findings [33].

Transcripts and observer notes were verified by the moderator and one of the observers. Deductive codes were based on the discussion guide questions. Inductive codes were based on themes identified in team debriefs after focus groups, as well as themes identified by the moderator during review of transcripts. All deductive and inductive codes were included in a standardized codebook, which was reviewed by several members of the team prior to their application to transcripts.

The moderator applied deductive and inductive codes to the transcripts using ATLAS.ti (version 6.2.28). The moderator then generated code reports, which were read for emergent themes that were common across groups, as well as unique and conflicting perspectives. Direct quotations from participants were used for support and illustrative purposes, and any content of quotes that was corrected for reader comprehension was included in square brackets. To minimize bias and include multiple perspectives of the key findings of the focus groups, all members of the data collection team reviewed the resulting report of findings and provided feedback.

## Results

### Participants

A total of 41 participants attended the six focus groups, with a range in focus group size from four (AR2) to 11 (OK1) participants. Focus groups lasted approximately 90 min.

#### Pre-focus group questionnaire results

From the 41 participants, 40 pre-focus group questionnaires were returned (98 % completion). Most of the participants were male (68 %) and older adults (68 %, ≥55 years of age). Only one participant (3 %) had children under the age of 2 years in the home, and 18 (45 %) had adults aged 65 years and older in the home. Most participants (75 %) had lived in their current residence and had used well water for more than 10 years.

Beyond domestic water uses, one person (3 %) used their well water for crop farming and seven (18 %) used it for animal farming. Almost all participants used their well water for washing dishes (98 %), bathing (98 %), cooking (95 %) and laundry (95 %), while 28 (70 %) used it for their drinking water. Half (53 %) of the participants had ever tested their water; testing was more common among participants from OK (77 %) and AR (50 %) as compared to IN (18 %). Of the 21 who tested, 11 (52 %) had done so more than 5 years ago. Eighteen participants (45 %) had checked their well depth; seven (39 %) of these had done so in the past 2 years. A change in odor (73 %), taste (70 %) and color (70 %) were the most common reasons for testing. Not knowing where to send water for testing was the most common reason (35 %) for not testing. Most participants wanted further information on what to test their well for (65 %) and when to test (55 %).

In Oklahoma, only one participant had heard of the OWRB’s Comprehensive Water Plan and no one had participated in a planning session.

### Focus group results

#### Severity of 2012 drought

Most participants in all groups indicated that the drought of 2012 was the most severe drought they had ever experienced. In OK, participants noted that local lakes had gone dry and water reserves were so low that some counties had to find additional sources of municipal water to pipe in for their residents. Mandatory water restrictions were in place in both counties, but participants in one group noted that the restrictions did not apply to private well owners since they maintain their own supply. In AR, several participants in both groups had been afraid that their wells would run dry. AR1 described trees dying, gardens and crops getting “burned up”, and extreme heat conditions.

*Moderator: How does this drought compare to other droughts that you’ve seen?*

*Participant 1: They’re all hot as the dickens.*

*Participant 2: Well, I’m probably older than anybody in here, and this was the biggest one I’ve seen. And when we was [kids], we farmed, and we called it share crop farming. And if it got dry, we was out of luck altogether. But this is the worst drought I’ve seen in my 85 years.*

*Participant 3: In’87 we had a pretty bad one, but it wasn’t nothing like last year.*

*(AR1)*

A few participants in both the IN groups debated whether the drought of 1988 may have been as severe as 2012.

*I mow yards for a living, so I kinda know. In’88 we would still get 1/10 of an inch here and there. This year, in July we didn’t have any precipitation whatsoever. It was just harder on everything.*

*(IN1)*

#### Well water quantity during 2012 drought

Though participants across all groups shared the perception that the drought was severe, few participants had seen changes in the quantity of their well water during the 2012 drought. With regard to a decrease in quantity, one participant from OK had seen his well water level decrease by five feet. Two other participants, one from AR and one from OK, had also seen a decrease in the amount of water in their wells during the drought; however, they thought that this decrease could have been attributed to other factors including a neighbor drilling a well close to their well and the nearby building of a neighborhood development with private wells, respectively.

In five of the six groups, at least one participant had heard of well owners whose wells had run dry during the drought, or they had heard of other areas in the state where wells had gone dry. While such reports were most common in OK1 and OK2, one participant from AR1 said he shared his well water with his neighbor whose well had gone dry.

#### Well water quality during 2012 drought

During the drought, some participants across the three states said they experienced a change in the quality of their water. In OK2, several participants noted they had more sand or sediment in their water. In AR2, most participants said they typically have mild discoloration of their water, but during the drought their water had turned browner or even black. Along with the discoloration, AR2 noted an increase in the intensity of the “sulfuric smell” of their water during the drought. In IN1, a participant noticed a change in the smell of the water and another participant said his neighbor’s water had a “sulfuric, rotten egg smell” during the drought.

*I ran a bath once, came back and looked at it, and the water was brown. I decided that I wasn’t as dirty as that water was.*

*(AR2)*

#### Options for a dry well

Because none of the participants’ wells had run dry, groups were asked what they would do if, hypothetically, their well were to go dry. The most common responses across all groups were to contact the local well driller and to drill a new well or deepen their existing well.

*About the only option would be to call a well guy and see if he could find some water.*

*(IN1)*

However, the cost of digging the well deeper was daunting to some participants, as illustrated in the discussion below.

*Participant 1: That first well I think I paid three hundred dollars to have it dug. That last one cost me two thousand dollars.*

*Participant 2: Yeah, it’s very expensive if you have to go deeper.*

*(AR2)*

Another common response to a hypothetical dry well was to seek out another water supply. Options here included more permanent changes such as connecting to public water supply, as well as temporary measures such as sharing from a neighbor’s well, purchasing bottled water, or, in the case of the IN focus groups, accessing surface water from one of many local lakes.

Although connecting to the public water supply was identified as a long-term solution to a well going dry, the idea evoked mixed reactions in participants. In AR, there have been concerted efforts to encourage well owners to transition to public water sources, and several of the participants there were more open to the idea of switching to public water. However, some were unable to do so because they lived too far from a water line or because the expense was too much of a barrier.

Many other participants were not open to connecting to a public water source. They were uninterested in connecting due to personal opinions about what they perceived to be the inferior taste and smell, health concerns, and the costs of connecting to and using municipal water.

*I don’t like drinking [city water] because of all the chemicals in it. It’s hardly fit to take a bath in, much less drink*

*(AR1)*

Several participants also said they would try to store water as a temporary response to a dry well. Participants suggested using a cistern or underground storage tank, or a pond or creek on their property. Some participants considered using rain barrels in the future to store water in case of drought, but they noted that by the time a drought was underway, there was no rain to fill the barrels.

OK1 also discussed the option of selling a house if the well went dry. They noted the impact of a dry well on property value. One participant indicated that a seller may be required to verify that the property’s well has worked properly for 1 year, and the seller would be responsible if a new well needed to be drilled within a given period of time after the purchase. Several participants indicated that they would be worried about selling their house if their well went dry, as the house would be “worthless”. One participant had already sold a house out of concern that the building of a nearby housing development that relied on wells would cause his well to go dry.

*[I would] put a “for sale” sign in the yard, and [maybe] get lucky and somebody [would] actually want to buy a house that didn’t have water.*

*(OK1)*

Finally, many participants across the six groups responded to the dry well scenario by shaking their head and shrugging their shoulders. They seemed to not know what they would do and to not have considered how they would respond to a dry well. One participant recommended that a well owner with a dry well should “pray like heck it’s going to rain.”

#### Response to 2012 drought

Participants identified several ways that they had tried to conserve water during the drought. These included indoor water use measures such as not letting the water run when brushing teeth or shaving, and outdoor measures of not watering the lawn as much or not at all, not watering gardens or plants, and not washing the car or washing it in an area served by municipal water. Some of the less common ways to conserve identified by participants included installing water-efficient toilets, using bottled water for brushing teeth, taking shorter showers, decreasing laundry frequency, and using pets’ leftover drinking water to water plants.

Conservation methods considered by participants but not yet implemented included installing a drip irrigation system for the garden, installing an on-demand hot water tank for showers, and using “gray water” for watering plants. In AR, some participants suggested storing well water in plastic containers for times of drought. Some in the group thought water stored in this way would “spoil” if stored too long and would become unsafe to drink, but it could still be used for flushing toilets. Others thought that if it looked clear, it would be safe to drink if boiled.

OK1 and OK2 raised concerns about neighbors not conserving water. Participants commented on observing neighbors using sprinklers wastefully (such as during the hottest times of the day), or using well water to irrigate the lawn of a golf course.

*I’ve got a neighbor…we’re on the same zone on good water. And he’s an okay guy, but there’s a set of sprinklers in his yard, a system, and they just go at 10, 11 in the daytime when it’s already 95° out. And they’re out there watering everywhere…it worries me. What do you do if you’ve got a neighbor who’s literally wasting water?*

*(OK2)*

Participants were also concerned that some nearby agricultural operations could be taxing their common groundwater supply. OK2 participants were surprised when one member said a local farming operation had begun to grow corn and expressed concern about the amount of water that this would use.

Participants in OK also suggested a variety of negative consequences to property, the economy, and health that could arise from excessive water conservation. One woman experienced property damage that she believed was indirectly due to the drought. By not watering around her house, she believed the dry ground led to shifting of her foundation, which then caused the pipes in her home to burst and flood the house. One participant was concerned about the fire risk to his property from the dry vegetation resulting from not watering, and from living in an area without municipal fire hydrants. One participant was worried that the local economy would suffer if businesses were forced to close or leave the area due to restrictions in water usage. Some thought that conserving water could have health impacts from poor hygiene, such as from bathing less often.

*Well other than quit watering my lawn and I don’t know of any other [conservation measures] that would be practical or healthy. You’ve got to look at health, and water is connected to people’s health a great deal. Our choices are very limited that’s for sure, very limited.*

*(OK2)*

#### Community of well owners

Across the six group sessions, there were common ways in which the participants interacted with one another. In each group there were some participants who had not had a well before and who asked the group for advice on how to address challenges with their well water. Some of the well owners who were more experienced with owning a well were very confident in their knowledge about wells and offered advice to newer well owners. As one example, a more experienced well owner provided his contact information to a less experienced well owner to be an on-going resource for that participant after the session.

#### Sharing information with well owners

Several participants expressed that they wanted to know more about what was happening in their community with regard to well owners. Participants in OK and IN wanted to receive notifications when wells in their area had issues, such as testing positive for contaminants, going dry, or causing diseases such as cancer. Several participants commented at the end of the sessions that they appreciated that the focus groups provided the opportunity to hear the questions and concerns of other well owners in their area.

In terms of information needs, only a few participants indicated that they wanted to know more about drought and well management in the context of a drought, and many participants indicated a need for more information about well water testing. Participants said they appreciated the information packages provided at the end of the sessions containing local, state and national resources on general well management.

Across groups, many participants indicated that U.S. mail and a hotline were good ways of conveying information to them. Participants in AR and IN also suggested public meetings as a good way to receive information about wells, and for opportunities to share concerns with other well owners. Few participants preferred email, and several participants indicated they did not have access to the internet, had a cumbersome, slow dial-up connection to the internet, or they did not like to use the internet.

## Discussion

The current study investigated the impact of drought on private well owners in three states in the U.S. The common themes that arose across focus groups may guide the efforts of the states, communities, and organizations that seek to support and sustain the 43 million private well owners in the U.S.

First, although participants acknowledged the severe drought conditions in 2012 as the worst drought they had experienced, few saw a decrease in the quantity of water in their well during the drought, although participants in each group had heard of other well owners who had wells that had run dry. In contrast, participants in each state observed changes to their water quality during the drought. Most water quality concerns were exacerbations of pre-existing quality issues of discoloration and “rotten egg” odors. Though these findings are promising as an indication that the drought may have had limited impact on the water supply of private well owners, further study using objective measures of water quality in future drought is warranted to further explore these findings.

Second, across groups it was clear that few participants had tested their water at recommended testing intervals. CDC recommends that private wells be tested yearly for mechanical problems, total coliform bacteria, nitrates, total dissolved solids, and pH [34]. Fewer than half of participants had ever tested their water, and of these, only half had tested in the last 5 years. These findings were consistent with other studies of well owners’ well testing behaviors, which have indicated that the majority of private well owners in the U.S. and Canada do not monitor their water quality for contaminants through recommended annual testing [35–38].

More than a third of participants indicated that their primary reason for not testing their water supply was not knowing where to send water samples, and many participants expressed interest in receiving additional information on what analytes to test for in their region, and when to test their wells. Well owners’ desire for more information about testing resources, locations, and procedures has been manifest in previous studies [36, 38]. At the end of each focus group, participants were provided packets of information on local testing resources, much of which was available on the internet. However, many well owners indicated that they were not comfortable using the internet to gain information about their wells or did not have good access to internet given that they lived in remote areas. This suggests that further outreach efforts to communicate with private well owners regarding private well testing could be effective if these efforts include traditional methods of communications (e.g., mailed newsletters, phone calls, community meetings, etc.).

Participants discussed various measures they had taken to reduce their water consumption during drought, with reduction of garden watering being the most common measure. The effect of these measures may have lessened the impact of the drought on their water supply, for example resulting in minimal water quantity concerns for participants. Various state and national level organizations provide information resources on water conservation for private well owners, including measures such as conducting a household water audit, using water-efficient technology, and changing water use behavior in the home and in the yard [39, 40]. While the focus groups often described changing the way they used water as a means of conserving water, few described using water-efficient technology or conducting household water audits. Further investigation is necessary to evaluate the risks and benefits of these various conservation measures to provide private well owners with additional guidance on which measures might be the most useful and cost effective.

All six of the groups discussed financial impact and cost-related factors associated with the potential impacts of drought. Many participants were concerned about the cost of having to deepen their well or drilling a new well if their well went dry. The issue of cost was most pronounced in OK, where participants discussed the link between the value of property and an adequate well water supply. In addition, participants discussed the connection fees and ongoing monthly fees associated with use of a municipal supply. The cost/benefits and potential financial burdens of staying on private wells, versus those associated with transitioning to a municipal water supply, serve as barriers and incentives in decision making and health behaviors of well owners. Another example of the impact of cost, or perceived cost, on well owners’ actions, is the finding that when well owners believe that water treatment is costly, it decreases the likelihood that they will treat their water [41]. Similarly, perceptions of the cost of testing and treating well water may influence testing behaviors. Future research may investigate the socio-economic status of private well owners and how the impact and perception of cost of water sources influences behaviors, such as testing behaviors and deciding to stay on private well water versus transition to a municipal water supply.

In addition to concern about cost, other common reasons for staying on private well water were the perceived inferior taste, smell, and possible adverse health effects from municipal water. In a study conducted in the United Kingdom and Portugal, researchers found that sensory properties of water, primarily flavor, most strongly influenced participants’ estimation of water quality [42]. The findings of our focus groups also support a strong preference for the taste and smell of private well water, which is consistent with previous research of well owners in Canada [36, 38]. In the current study, changes to the sensory properties of private well water were primary reasons for testing well water; changes in odor (73 %), taste (70 %) and color (70 %) were the most common reasons for testing. The tangible, sensory qualities of well water are, therefore, key to the appeal of well water as well as triggers for concern about health risks posed by well water. However, it should be noted that many chemical and microbial contaminants are tasteless and odorless; thus, well owners could benefit from further education that emphasizes the need to test well water in the absence of any foul taste or smell.

Both the historical context of drought and the geological context of the three states appeared to have influenced participants’ perceptions of drought and created some variation in findings between states. For example, both OK and AR regularly experience hot summers and drought conditions. While participants agreed that 2012 was worse than other summers, they felt that they routinely try to conserve water and had difficulty thinking of additional water conservation measures they could implement.

In comparison, the 2012 drought in IN was an unusual event, and participants commented that they were more used to dealing with flooding and heavy rains. IN1 and IN2 did not report as many water conservation efforts as did participants in AR and OK.

As another example of regional differences that may have impacted findings, the AR groups indicated that the government had made efforts to make the public water supply available to private well owners, and several in the groups wanted to connect to this supply but could not due to distance from the water lines or great cost of connecting. Several members of these groups would have liked to connect to the public water supply, whereas most members of the OK groups were strongly opposed to connect to public water and were proud of their well water, feeling that it was superior to public water in taste and a better financial decision. Sensitivity to the differences that geography and political history can create in the experience and behaviors of private well owners will be important in future research and outreach. As governments, communities, and organizations develop tools and approaches to better serve private well owners, it is recommended that they conduct assessments to determine the unique needs and perceptions of local well owners.

Research on the impacts of drought has identified social networks as having an important role in mitigating the effects of these slow-onset disasters [8–10]. Several themes of community emerged from the six focus group sessions. Some groups discussed the supportive role of neighbors in sharing their well water supply when wells went dry. Other groups identified a breakdown of ‘neighborliness’ when participants felt uneasy about what they saw as excessive uses of water by their neighbors. Some participants seemed to value a sense of social cohesion that even though households had their own private well supply, they were connected to the same groundwater supply, and every household had to be responsible for sharing it. Following from this notion, some participants suggested publicly posting (de-identified) information on wells so that neighbors can be aware of any concerns in their area. Each focus group also became its own social network where participants began to meet neighbors they had not known before, and more experienced well owners offered advice and support to those who were new to owning a well. The pre-focus group questionnaire asked participants where they look for information on wells and 11 (28 %) said they did not look for information on well management. However, the positive response at the end of each session suggests that these types of groups may promote valuable social networks.

### Limitations

There are several potential limitations to this study. With the majority of participants being English-speaking older adult males, the experiences of females, younger adults, families with small children, and immigrants were not as well represented in the results, and the findings may not be readily transferrable to these populations. In addition, given the mention of the financial implications of decisions about well water, socio-economic status may have influenced behaviors and perceptions of well owners, however no data were collected on participants’ socio-economic status.

Differences in the weather in each location at the time of the focus groups may have heightened participant responses in OK and tempered them in IN and AR. At the time of the focus groups in AR and IN (April 2013), the areas were experiencing regular rains and thunderstorms. In comparison, OK1 and OK2 (March 2013) occurred when there was on-going news of low water levels in reservoir lakes. Some participants in OK were still actively considering options for their response to drought, while participants in IN generally had no specific plans for future droughts. A previous study on the timing of surveys of water conservation found that attitudes towards conservation were heightened during dry seasons compared to wet seasons [43].

Participants were asked during focus group sessions to describe their well systems as a means to initiate dialogue amongst participants, crudely assess their level of understanding about their system, and identify any significant differences in well systems that may impact water quality/quantity. Participants within a focus group that were aware of their system had similar well depths and number of wells. However, as this information was not systematically collected or corroborated, findings were not included in this analysis, but should be considered in future studies.

Despite several efforts to remind those who agreed to participate to show up to the session, not all focus groups achieved their target size of six to ten participants. The fact that the Oklahoma groups were the largest of the focus groups may reflect the greater concern of well owners in that area to the persistent drought. One of the goals of focus groups as a qualitative research method is to stimulate dialogue among participants to achieve richer results than can be achieved by individual interviews. Regardless of the number of participants in the focus groups, each group provided rich results that added to the overall analysis. Similar themes and group dynamics were apparent from each of the sessions, suggesting that smaller group sizes did not significantly impact the quality of results from smaller sessions.

Conducting six focus groups, two counties per state in three states, provided a diverse range of experiences of participants. While there were many common themes, each session also provided county and state-specific information. This suggests that conducting more focus groups in other states that experienced drought in 2012 may provide additional results that have not been identified by this investigation. However, where common themes did emerge, there was strong consistency across the six focus groups, supporting the validity and robustness of the data and the transferability of these common themes.

## Conclusions

As far as we are aware, this is the first investigation of the public health impacts of drought on private well owners. Our findings provide a rich understanding of the issues and concerns of private well owners in the U.S. in terms of their overall well ownership, as well as their experiences during the 2012 drought.

Across focus groups, there was evidence that private well owners do not test their water supply regularly, and they want more information and notifications about well water testing. Governments, communities, and organizations that seek to support private well owners may focus on making testing resources accessible and ensuring awareness of available testing resources through outreach in plain language.

Findings also highlighted the variation in well owners’ perceptions and experiences due to historic and geographic differences. Therefore, ongoing qualitative and quantitative assessment are important to understanding the unique experience and context of well owners, particularly as they may inform the development of policy, interventions, and communications with private well owners.

Through ongoing qualitative and quantitative inquiry of the needs of private well owners, the safety of the water supply of these millions of Americans may be continually monitored, improved, and secured, thus supporting the goal of a safe and adequate water supply for all Americans.

### Ethics approval and consent to participate

The study protocol including processes for obtaining and managing informed consent from participants was reviewed by the CDC NCEH/ATSDR Human Subjects Contact office and the Oklahoma State Department of Health Institutional Review Board and determined exempt from full board and continuing review.

### Consent for publication

Not applicable.

### Availability of data and materials

The dataset supporting the conclusions of this article is included within the article. Additional details on specific focus group transcript information may be available upon request and with permission from the authors.

## Abbreviations

AR: Arkansas
AWWA: American Water Works Association
CDC: Centers for Disease Control and Prevention
EPA: Environmental Protection Agency
IN: Indiana
NOAA: National Oceanic and Atmospheric Administration
OK: Oklahoma
OWRB: Oklahoma Water Resources Board
US: United States
USGS: US Geological Survey
